# Comparison of HbA1c and OGTT Results in Obese and Morbidly Obese Patients: A Single-Center Evaluation From an Obesity Clinic

**DOI:** 10.1155/jdr/9933219

**Published:** 2025-09-04

**Authors:** Banu Açmaz, Sami Bahçebaşı, Erdem Aydın, Şeyma Yıldız, İrem Biçer

**Affiliations:** Department of Internal Medicine, Kayseri City Training and Research Hospital, Kayseri, Türkiye

**Keywords:** diabetes mellitus, insulin, obesity, OGTT

## Abstract

**Introduction:** Obesity, particularly morbid obesity, is associated with significant metabolic alterations such as insulin resistance and compensatory hyperinsulinemia. These pathophysiological changes may influence the performance and interpretation of glycemic diagnostic tools, including the oral glucose tolerance test (OGTT) and glycated hemoglobin (HbA1c). This study is aimed at comparing the diagnostic profiles of OGTT and HbA1c across BMI categories, with a specific focus on morbid obesity and the potential role of elevated insulin levels in test discrepancies.

**Materials and Methods:** This retrospective study included 1031 patients who were classified into nonobese, obese, and morbidly obese groups based on body mass index (BMI). All participants underwent assessment of glycemic status, lipid profile, and related metabolic parameters.

**Results:** Of the study population, 86.8% were female. Based on BMI classification, 56.5% were obese, 35.8% morbidly obese, and 7.8% nonobese. The prevalence of dysglycemia (prediabetes + diabetes) was 43.1% according to HbA1c and 30.9% according to OGTT. Dysglycemia was significantly more frequent in the morbidly obese group by HbA1c (*p* < 0.001), while OGTT showed no significant intergroup difference (*p* = 0.117). The agreement between methods was low (*κ* = 0.326). HbA1c, insulin, HOMA-IR, CRP, and triglyceride levels increased with BMI. Insulin-related parameters were higher among HbA1c-defined dysglycemic individuals.

**Discussion:** HbA1c may be more sensitive in identifying early metabolic disturbances, particularly during the compensatory phase involving hyperinsulinemia. Elevated insulin levels may attenuate glucose response during OGTT, potentially reducing its diagnostic sensitivity.

## 1. Introduction

Obesity is defined as the excessive accumulation of fatty tissue, which is associated with an increased risk of adverse health outcomes. However, the manifestation of these health problems is not a necessary condition for the diagnosis of obesity [1]. Obesity classification primarily relies on the measurement of body mass index (BMI), which is calculated by dividing a person's weight (in kilograms) by the square of their height (in meters) (kilogram per square meter). A BMI value of ≥ 25 kg/m^2^ classifies an individual as overweight, while a BMI of ≥ 30 kg/m^2^ indicates obesity [2]. Regardless of the definition, it is well-established that obesity is strongly linked to an increased risk of Type 2 diabetes mellitus, depression, cardiovascular diseases, certain cancers, and higher mortality rates. Furthermore, it is projected that by 2030, 85% of adults will be overweight or obese [3].

When obesity develops, metabolic morbidity increases; however, this situation has no validity for all cases. Thus, obese patients, who are complicated with abnormal metabolism, have a great tendency to develop metabolic disorders; on the other hand, very little proportion of these patients have normal metabolism [4]. Type 2 DM prevalence is increasing, and pathogenesis depend on both environmental and genetic factors associated with increased hepatic glucose synthesis, disordered insulin secretion and resistance to insulin. Impaired glucose and insulin metabolism has typically close bound with obesity. Not all but most of the Type 2 DM patients are obese at the beginning point of disease [5].

In this study, patients who were admitted our obesity clinic for treatment were investigated. These patients were separated into groups according to obesity classification system (normal overweight, obese, and morbid obese) and matched for metabolic abnormalities. Moreover, we aimed to investigate prevalence of prediabetes, Type 2 DM, and hyperlipidemia among these patients.

## 2. Materials and Methods

### 2.1. Study Design and Population

This retrospective study was conducted between December 2023 and May 2024 at the Obesity Outpatient Clinic of the Department of Internal Medicine, Kayseri City Hospital. The study was approved by the Institutional Ethics Committee of Kayseri City Hospital (Approval No: 12.12.2023-988). A total of 1943 patient records were screened, and 1031 eligible volunteers, who met the inclusion criteria and were admitted to the Obesity Clinic, were included in the final analysis. Age, sex, BMI, obesity classification, and metabolic status were recorded for each participant. The relationship between BMI and metabolic status was also evaluated.

All patients underwent a detailed physical examination performed by the same investigator (B.A.). Body weight was measured using a Tanita scale while patients wore only underwear and were barefoot. Height was measured using a wall-mounted stadiometer. BMI was calculated using the standard formula: weight (kilogram)/height^2^ (square meter).

Initial classification was based on standard BMI thresholds defined by international guidelines as follows:

1. BMI 18.0–24.9 kg/m^2^: normal weight
2. BMI 25.0–29.9 kg/m^2^: overweight
3. BMI 30.0–39.9 kg/m^2^: obese
4. BMI ≥ 40.0 kg/m^2^: morbidly obese [6, 7]

For statistical analysis, patients were regrouped into three BMI categories:

1. Nonobese group (*n* = 80): patients with BMI < 30.0 kg/m^2^, including both normal weight and overweight individuals.
2. Obese group (*n* = 582): patients with BMI between 30.0 and 34.9 kg/m^2^.
3. Morbidly obese group (*n* = 369): patients with BMI ≥ 35.0 kg/m^2^.

Exclusion criteria are as follows:

1. Presence of thyroid disorders or current use of thyroid medications
2. Use of lipid-lowering agents
3. Use of estrogen, corticosteroids, narcotics, insulin, or barbiturates
4. History of diabetes mellitus or hyperlipidemia

Inclusion criteria are as follows:

1. Admission to the obesity clinic due to overweight or obesity

## 3. Admission for Weight Control or Lifestyle Counseling

### 3.1. Diabetes Screening Algorithm

Asymptomatic individuals with a BMI ≥ 25 kg/m^2^ (or > 23 kg/m^2^ for Asian populations) and at least one additional risk factor should be screened for diabetes at an earlier age and more frequently (e.g., annually). These risk factors include a family history of diabetes, high-risk ethnic background, history of macrosomic birth (≥ 4 kg) or prior gestational diabetes, hypertension, dyslipidemia, clinical conditions or physical signs associated with insulin resistance (e.g., acanthosis nigricans), history of cardiovascular disease, low birth weight, sedentary lifestyle, solid organ transplantation, long-term corticosteroid or antiretroviral therapy, and use of antipsychotic medications.

According to the American Diabetes Association (ADA) Standards of Medical Care in Diabetes—2023, blood samples during the OGTT should be collected within a ± 5-min time window, and plasma glucose levels should be measured in venous samples. Prior to testing, participants should consume at least 150 g of carbohydrates daily for a minimum of 3 days, maintain an overnight fast of at least 8 h, and avoid excessive physical activity and alcohol intake [8].

In our study, patients who underwent OGTT were informed and prepared in accordance with the ADA 2023 guidelines that were in effect at the time of their admission. Venous plasma samples were used for all glucose measurements.

The first-line screening tests for diabetes are fasting plasma glucose and HbA1c. Individuals with a fasting glucose level < 100 mg/dL or HbA1c < 5.7% who have one or more additional risk factors should undergo a 75-g OGTT. Fasting glucose levels between 100 and 125 mg/dL or HbA1c values between 5.7% and 6.4% also indicate the need for OGTT in this population. Based on OGTT results, individuals are diagnosed with prediabetes (isolated IFG, isolated IGT, or both) or diabetes and are followed accordingly. If OGTT results are normal, periodic rescreening is recommended. Although most international guidelines suggest screening every 3 years, more frequent testing—preferably annually—may be appropriate for individuals with significant weight gain or multiple metabolic risk factors [9].

We performed OGTT on patients who were evaluated in our clinic and were not previously diagnosed with diabetes. For the test, blood is taken for basal values after 8 h of fasting, and then, blood is taken from the vein in the antecubital area at the second hour after drinking 75 g of glucose. As a result of OGTT, blood sugar measurement at the zeroth hour is ≥ 126 mg/dL and blood sugar measurement at the second hour is ≥ 200 mg/dL, which establishes the diagnosis of diabetes mellitus.

Isolated impaired fasting glucose (IFG) was defined as fasting plasma glucose between 100–125 mg/dL, while isolated impaired glucose tolerance (IGT) was defined as 2-h plasma glucose between 140 and 199 mg/dL. When both values fell within these ranges simultaneously, a diagnosis of IFG + IGT or prediabetes was made [8, 10].

Dysglycemia is a broad term encompassing all abnormalities in glucose metabolism, including both prediabetes (IFG, IGT, or HbA1c 5.7%–6.4%) and overt or newly diagnosed diabetes. According to current clinical guidelines, dysglycemia represents a spectrum of glycemic disturbances ranging from early insulin resistance to frank hyperglycemia exceeding diagnostic thresholds [11].

In this study, all individuals identified as having either prediabetes or diabetes based on HbA1c or OGTT results were classified under the dysglycemia group.

Insulin resistance was calculated using the HOMA-IR formula: [fasting insulin (*μ*U/mL) × fasting glucose (mg/dL)]/405. A threshold of HOMA − IR > 2.5 was considered indicative of insulin resistance [12].

### 3.2. Statistical Analyzes

All statistical analyses were performed using IBM SPSS Statistics for Windows, Version 22.0 (IBM Corp., Armonk, NY, United States). The normality of distribution was assessed using the Shapiro–Wilk test, and homogeneity of variances was evaluated with the Levene test. As most continuous variables were not normally distributed, nonparametric tests were applied. Continuous variables were expressed as median (25th–75th percentile).

Kruskal–Wallis *H* tests were used to compare differences between BMI-based groups, and Mann–Whitney *U* tests were applied for pairwise comparisons when necessary. Effect size estimates (*ε*^2^) were calculated for Kruskal–Wallis tests to enhance interpretability. Spearman correlation analysis was used to assess the relationship between 2-h OGTT glucose values and fasting insulin or HOMA-IR levels.

Cross-tabulation analyses were performed to evaluate the distribution of glycemic status across BMI categories. The Chi-square test was used to compare categorical variables. In addition, the McNemar test was conducted to assess the concordance between diabetes and prediabetes diagnoses made by HbA1c and 75-g OGTT. A *p* value < 0.05 was considered statistically significant for all tests.

## 4. Results

A total of 1031 patients were included in the study, of whom 86.8% were female (*n* = 895) and 13.2% were male (*n* = 136). Based on BMI classification, 56.5% were obese (*n* = 582), 35.8% were morbidly obese (*n* = 369), and 7.8% were nonobese (*n* = 80). The prevalence of dysglycemia (prediabetes + diabetes) was 43.1% (*n* = 444; prediabetes: *n* = 410 and diabetes: *n* = 34) according to HbA1c, and 30.9% (*n* = 319; prediabetes: *n* = 269 and diabetes: *n* = 50) according to OGTT.

Analyses conducted across BMI categories revealed significant elevations in HbA1c, ALT, CRP, insulin, HOMA-IR, triglycerides, and age with increasing BMI (*p* < 0.001 for all). FPG levels were significantly lower in the obese group compared to both nonobese and morbidly obese individuals, whereas 2-h PG levels were lowest in the nonobese group. HDL-C levels were significantly reduced in the obese and morbidly obese groups relative to the nonobese group (*p* < 0.001).

Despite the relatively small sample size in the nonobese group, the consistency of intergroup differences suggests a robust association between BMI and these metabolic parameters. Effect size estimates further support the clinical relevance of these findings. Complete data are presented in Table 1.

**Figure 1:**
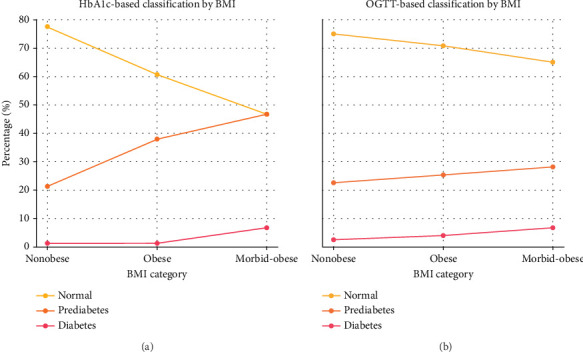
Distribution of glycemic categories by BMI based on HbA1c and OGTT. (a) Distribution of normal glycemia, prediabetes, and diabetes prevalence according to HbA1c criteria across the nonobese, obese, and morbidly obese groups. (b) Corresponding distribution based on OGTT criteria in the same BMI categories.

As shown in Table 2, the distribution of glycemic categories differed significantly across BMI groups when classified by HbA1c (*p* < 0.001), whereas OGTT-based categories did not show a statistically significant difference (*p* = 0.117). According to HbA1c, the proportion of patients diagnosed with diabetes increased with higher BMI, from 1.3% in the nonobese group to 6.8% in the morbidly obese group. Prediabetes rates also rose markedly, reaching 46.6% in the morbidly obese group. In comparison, OGTT identified diabetes in 2.5% of nonobese, 4.0% of obese, and 6.8% of morbidly obese individuals, with more modest variation across BMI categories. The graphical distribution of glycemic categories by diagnostic method and BMI classification is presented in Figure 1.

**Figure 2:**
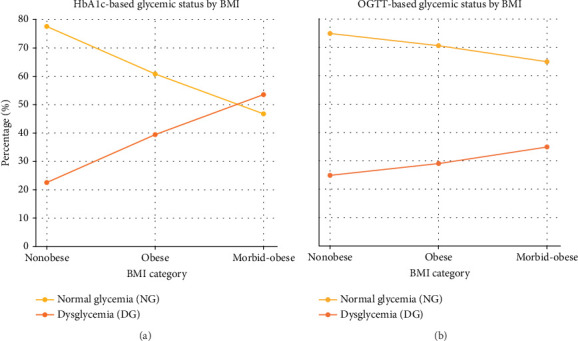
Normal glycemia and dysglycemia by BMI category. (a) Proportion of individuals with normal glycemia (NG) and dysglycemia (DG) based on HbA1c criteria in the nonobese, obese, and morbidly obese groups. (b) Corresponding distribution using OGTT criteria across the same BMI categories.

When DM and pre-DM were grouped together as dysglycemia, an increasing prevalence of dysglycemia with higher BMI was observed across both diagnostic methods. The comparative proportions of normoglycemia and dysglycemia by the BMI group are illustrated in Figure 2.

Based on HbA1c, dysglycemia rates rose from 22.5% in the nonobese group to 53.4% in the morbidly obese group (*p* < 0.001). A similar trend was noted with OGTT (25.0%–35.0%), although the difference did not reach statistical significance (*p* = 0.085). McNemar's test revealed a statistically significant discrepancy between the diagnostic outcomes of HbA1c and OGTT in the same individuals (*χ*^2^ = 46.7, *p* < 0.001). The Cohen's kappa coefficient was 0.326 (*p* < 0.001), indicating only fair agreement between the two tests. The detailed results are presented in Table 3.

When BMI groups were evaluated separately according to glycemic status based on HbA1c, FPG and 2-h PG levels were significantly higher in dysglycemic individuals across all categories (*p* < 0.001 for each). Among obese and morbidly obese individuals, insulin and HOMA-IR values were also significantly elevated in the dysglycemic group (*p* = 0.037 and *p* < 0.001 in obese; *p* = 0.019 and *p* < 0.001 in morbidly obese). In the nonobese group, despite significant differences in glucose levels, no meaningful differences were observed in insulin or HOMA-IR. CRP levels were higher in dysglycemic individuals in both the obese (*p* = 0.042) and morbidly obese (*p* = 0.046) groups. BMI did not differ significantly between normoglycemic and dysglycemic individuals within any group. These findings are presented in Table 4.

## 5. Discussion

Obesity and Type 2 DM share many common pathophysiological mechanisms, and the prevalence of both conditions is steadily increasing worldwide. Therefore, screening for Type 2 DM in individuals with obesity is considered a more effective strategy compared to screening in the general population [13]. In this study, dysglycemia screening results of patients who presented to an obesity center were retrospectively evaluated.

In our study, insulin levels were significantly higher in morbidly obese individuals compared to obese individuals and in obese individuals compared to nonobese individuals. Similarly, HbA1c levels were higher in the morbidly obese group than in the obese group, and higher in the obese group than in the nonobese group. Additionally, CRP levels were found to increase significantly in parallel with higher BMI and insulin resistance. This finding supports the pathophysiological relationship between obesity-related chronic inflammation and metabolic syndrome. Considering that metabolic syndrome is regarded as a clinical manifestation of insulin resistance, the increase in CRP levels may indirectly reflect progressive metabolic deterioration in individuals with higher degrees of obesity [14].

Triglyceride levels were higher in the morbidly obese group, while HDL-cholesterol levels were lower. These metabolic alterations are thought to reflect either the effect of insulin or the degree of insulin resistance in these individuals. It is known that elevated lipid levels do not influence hepatic glucose production or ATP synthesis in muscle due to insulin resistance [15]. Therefore, increased glucose and insulin levels may create an abnormal metabolic environment that favors fat storage. A magnetic resonance spectroscopy study in the literature demonstrated that insulin resistance leads to ectopic fat accumulation in the liver and muscles [16].

Expansion of ectopic fat tissue, insulin resistance, and low-grade systemic inflammation contribute to the rapid deterioration of *β*-cell function and a gradual rise in blood glucose levels [17]. As insulin resistance progresses, glucose uptake in muscle tissue further decreases, leading to elevated postprandial plasma glucose levels and excessive insulin secretion. However, before overt diabetes develops, this hyperinsulinemia may maintain fasting plasma glucose levels within the normal range. During this compensatory hyperinsulinemic phase, hepatic glucose production is suppressed and glucose tolerance initially remains intact, yet postprandial glucose levels may progressively rise due to the limited capacity for glucose uptake in muscle tissue [18, 19].

However, the duration between the end of the compensatory phase and the onset of overt dysglycemia, as well as the extent to which metabolic abnormalities can be detected during this transitional period, remain unclear. Moreover, the effects of hyperinsulinemia on fasting and postprandial glucose levels during this phase are not well-defined. In our study, obese patients without known dysglycemia were screened from this perspective, and dysglycemia rates were found to be significantly higher in the morbidly obese group when assessed using HbA1c. In contrast, no significant difference was observed between groups when using OGTT. This may be explained by the increased insulin resistance associated with higher body weight, where elevated insulin levels during OGTT might reduce glucose levels and thus lower the sensitivity of the test. HbA1c, which reflects a longer term glycemic status, may be a more sensitive tool for detecting dysglycemia during this ambiguous transitional period, in which the effects of compensatory mechanisms and hyperinsulinemia are less apparent. Our analysis also showed that the higher prevalence of dysglycemia was mainly due to increased rates of prediabetes, and HbA1c appeared to be more sensitive in detecting this condition. The predominance of prediabetic individuals in our cohort suggests that many patients may be in this intermediate phase, where pathophysiological changes are not yet fully established.

In all BMI categories, individuals classified as dysglycemic based on HbA1c had significantly higher fasting and 2-h plasma glucose levels compared to normoglycemic individuals. This finding suggests that the two tests support each other in identifying glycemic abnormalities. Although fasting insulin and HOMA-IR levels were also higher in the dysglycemic groups, this difference reached statistical significance only in the obese and morbidly obese groups, indicating that insulin resistance becomes more pronounced with increasing adiposity. CRP levels, a marker of systemic inflammation, were also elevated in dysglycemic individuals, particularly in the morbidly obese group. Interestingly, within each BMI category, there was no significant difference in BMI between normoglycemic and dysglycemic individuals, which further highlights the role of insulin resistance in the development of dysglycemia independent of BMI itself.

Although HbA1c offers logistical advantages in clinical practice, it may be influenced by conditions unrelated to glycemic status, such as iron deficiency anemia or hemoglobinopathies. Furthermore, OGTT is still considered the gold standard for diagnosing dysglycemia according to current ADA guidelines. In this real-world data-based study, dysglycemia was more frequently detected using HbA1c, particularly in morbidly obese individuals. This may reflect the increased metabolic burden and the effects of insulin-mediated glucose suppression. However, these mechanistic explanations remain hypothetical, and prospective studies are needed to establish causality.

## 6. Conclusion

Many patients present with the expectation of weight loss; the frequent detection of previously unrecognized metabolic conditions such as dysglycemia is striking. Obesity is a serious disease associated with increased mortality and widespread metabolic dysfunction. Given the difficulty of achieving lasting treatment once obesity is established, early education on healthy lifestyle habits remains the most effective preventive strategy. Early detection and regular monitoring of dysglycemia are essential—not only to prevent complications but also to reduce obesity-related mortality. Therefore, routine screening with HbA1c, especially in individuals with obesity, should be prioritized, and more comprehensive studies are needed to compare diagnostic methods and improve their specificity.

### 6.1. Limitations

This study has several limitations. First, due to its retrospective design, timed insulin measurements were not available, which precluded dynamic analyses such as insulin curve or area under the curve (AUC). Additionally, cost-effectiveness analysis comparing HbA1c and OGTT was not performed; thus, conclusions regarding the economic utility of each method remain speculative. Body composition parameters such as waist circumference or body fat percentage were also not recorded, limiting deeper insights into fat distribution. Furthermore, the sample was predominantly female, which may affect generalizability. Despite these limitations, the study provides clinically relevant insights into the diagnostic performance of HbA1c and OGTT across obesity categories.

## Figures and Tables

**Table 1:** Comparison of metabolic and biochemical parameters according to BMI categories.

**Variables**	**Nonobese ** **(** **n** = 77**)**	**Obese ** **(** **n** = 572**)**	**Morbid obese ** **(** **n** = 362**)**	**p** ** value**	**Epsilon-squared ** **(** **ε** ^2^ **)**
Age	34 (25–42)	36 (27–44)	40 (29–49)	**< 0.001**	0.0197
FPG	92 (86–95)^a^	90 (84–97)^b^	92 (86–98)^a^	**0.023**	0.0055
2-h PG	105 (86–118)^a^	109 (88–128)^ab^	108 (90–135)^b^	**0.048**	0.0040
HbA1c	5.5 (5.2–5.6)	5.50 (5.3–5.8)	5.7 (5.4–6)	**< 0.001**	0.0455
eGFR	114 (103–121)	112 (102–122)	111 (99–120)	0.163	0.0016
AST	18 (15–22)	18 (15–22)	18 (15–22)	0.223	0.0010
ALT	17 (12–22)	18 (14–25)	19.67 (15–27)	**< 0.001**	0.00089
CRP	2.8 (0.9–3.8)	3.6 (1.9–6.6)	6.4 (3.8–11.1)	**< 0.001**	−0.0011
Insulin	14 (7.5–13.8)	14.8 (10.3–19.2)	17.3 (12.6–24.3)	**< 0.001**	−0.0014
HOMA	2.4 (1.7–3.2)	3.1 (2.3–4.3)	3.9 (2.8–5.8)	**< 0.001**	0.0206
TC	182 (160–204.5)	185 (162–209)	182 (160–210.5)	0.644	0.0281
LDL-C	120 (95–137.5)	119 (99–141)	116 (98–140)	0.735	0.1305
TG	104 (76–135)	127 (96–176)	134 (99.5–176)	**< 0.001**	0.0749
HDL-C	53 (45.5–60)^a^	47 (41–55)^b^	45 (39–52)^b^	**< 0.001**	0.0710

*Note:* Data are expressed as median (25th–75th percentile). . Superscript letters (a, b, c) indicate statistically significant pairwise differences between groups based on nonparametric tests (Kruskal–Wallis followed by pairwise Mann–Whitney *U* tests, *p* < 0.05). Effect size was calculated using epsilon-squared (*ε*^2^) for Kruskal–Wallis tests. Values of 0.01, 0.06, and 0.14 correspond to small, medium, and large effects, respectively. Bold values indicate statistical significance (*p* < 0.001).

Abbreviations: 2-h PG, 2-h plasma glucose (measured during oral glucose tolerance test); ALT, alanine aminotransferase; AST, aspartate aminotransferase; CRP, C-reactive protein; eGFR, estimated glomerular filtration rate; FPG, fasting plasma glucose; HbA1c, glycated hemoglobin; HDL-C, high-density lipoprotein cholesterol; HOMA, homeostasis model assessment for insulin resistance; LDL-C, low-density lipoprotein cholesterol; OGTT, oral glucose tolerance test; TC, total cholesterol; TG, triglycerides.

**Table 2:** Comparison of HbA1c-based and OGTT-based diagnostic categories by BMI group.

	**HbA1c**				**OGTT**			
	**NG ** **(** **n** = 587**)**	**PreDM ** **(** **n** = 410**)**	**DM ** **(** **n** = 34**)**	**p**	**NG ** **(** **n** = 712**)**	**PreDM ** **(** **n** = 269**)**	**DM ** **(** **n** = 50**)**	**p**
Nonobese (*n* = 80)	62 (77.5%)	17 (21.3%)	1 (1.3%)		60 (75.0%)	18 (22.5%)	2 (2.5%)	
Obese (*n* = 582)	353 (60.7%)	221 (38.0%)	8 (1.4%)		412 (70.8%)	147 (25.3%)	23 (4.0%)	
M-obese (*n* = 369)	172 (46.6%)	172 (46.6%)	25 (6.8%)	< 0.001	240 (65.0%)	104 (28.2%)	25 (6.8%)	0.117

*Note:* Comparison of diagnostic distributions based on HbA1c and OGTT across BMI groups. Each cell presents the count and percentage of patients within the BMI group diagnosed as normal, prediabetic (PreDM), or diabetic (DM) according to the respective method. Chi-square test results are included for each method.

**Table 3:** Distribution of normoglycemia and dysglycemia by BMI category based on HbA1c and OGTT and diagnostic concordance between the two methods.

	**NG (HbA1c) ** **(** **n** = 587**)**	**DG (HbA1c) ** **(** **n** = 444**)**	**p**	**NG (OGTT) ** **(** **n** = 712**)**	**DG (OGTT) ** **(** **n** = 319**)**	**p**
Nonobese (*n* = 80)	62 (77.5%)	18 (22.5%)		60 (75.0%)	20 (25.0%)	
Obese (*n* = 582)	353 (60.7%)	229 (39.3%)		412 (70.8%)	170 (29.2%)	
Morbid-obese (*n* = 369)	172 (46.6%)	197 (53.4%)	**< 0.001**	240 (65.0%)	129 (35.0%)	0.085

*Note:* McNemar *χ*^2^(1) = 46.7, *p* < 0.001; Cohen's *κ* = 0.326, *p* < 0.001. HbA1c and OGTT were used to classify glycemic status across BMI categories. Statistical significance for group comparisons was assessed using the Chi-square test. Concordance between HbA1c and OGTT was evaluated using McNemar's test and Cohen's kappa coefficient. Bold values indicate statistical significance (*p* < 0.05).

Abbreviations: DG, dysglycemia; NG, normoglycemia.

**Table 4:** Comparison of fasting insulin, HOMA-IR, and related metabolic markers between normoglycemic and dysglycemic individuals (based on HbA1c), by BMI category.

**Nonobese (** **n** = 80**)**	**Normoglycemia (** **n** = 62**)**	**Dysglycemia (** **n** = 18**)**	**p**
FPG	89.5 (84.5–94.5)	96 (94–102)	**< 0.001**
2-h PG	98 (80.5–111.5)	126 (99–143)	**0.004**
CRP	2 (0.9–3.7)	1.9 (1.4–4.2)	0.763
Insulin	11.2 (7.4–14.1)	11.1 (8.8–13.8)	0.553
HOMA	2.3 (1.6–3)	2.6 (2–2.3)	0.195
BMI	28 (27–29)	28 (27–28.5)	0.995
Obese (*n* = 582)	Normoglycemia (n = 353)	Dysglycemia (*n* = 229)	p
FPG	89 (83–94)	94 (88–102)	**< 0.001**
2-h PG	98 (82–118)	119 (102–144)	**< 0.001**
CRP	3.6 (1.7–6.3)	4 (2.1–7.2)	**0.042**
Insulin	13.5 (10.2–18.3)	14.4 (14.4–10.4–20.4)	**0.037**
HOMA	2.9 (2.2–4)	3.4 (2.4–5)	**< 0.001**
BMI	35 (32–37)	35 (33–37)	0.623
M-obese (369)	Normoglycemia (172)	Dysglycemia (*n* = 197)	p
FPG	89 (83–93)	95 (89–105)	**< 0.001**
2-h PG	102 (85–119)	120 (97–151)	**< 0.001**
CRP	6.9 (3.5–10.7)	16.9 (4.1–11.4)	**0.046**
Insulin	16.8 (11.8–21.4)	18 (13.3–27.2)	**0.019**
HOMA	3.6 (2.5–4.6)	4.3 (3–6.6)	**< 0.001**
BMI	43 (41–44)	44 (41–47)	0.051

*Note:* Values are presented as median (25th–75th percentile). Statistical comparisons between the normoglycemic and dysglycemic groups were performed using the Mann–Whitney *U* test. Bold values indicate statistical significance (*p* < 0.05).

Abbreviations: 2-h PG, 2-h plasma glucose; BMI, body mass index; CRP, C-reactive protein; FPG, fasting plasma glucose; HOMA-IR, homeostasis model assessment of insulin resistance.

## Data Availability

The data that support the findings of this study are available from the corresponding author upon reasonable request.

## General Statement

***What Is Already Known on This Topic.*** When obesity develops, metabolic morbidity increases; however, this situation has not validity for all cases. Type DM screening tests are required in patients with obesity. ***What This Study Adds.*** In obese patients, the HbA1c test may be more useful than the 75 g OGTT. Additionally, HbA1c is easy to apply and does not require a fasting state. ***How This Study Might Affect Research, Practice,* or *Policy*:** HbA1c test may be first step screening test in clinical practice.

## Supporting Information

Supporting Information Additional supporting information can be found online in the Supporting Information section. The supporting file contains the original SPSS output files of all statistical analyses performed in the study. These files were used to generate the tables and figures presented in the main manuscript.
