# Supplementary material for: Comparison of HbA1c and OGTT Results in Obese and Morbidly Obese Patients: A Single-Center Evaluation From an Obesity Clinic
Source: J Diabetes Res. 2025 Sep 4;2025:9933219. doi: 10.1155/jdr/9933219 (PMC12425632; doi:10.1155/jdr/9933219)
Supplement: Supporting Information — Additional supporting information can be found online in the Supporting Information section. The supporting file contains the original SPSS output files of all statistical analyses performed in the study. These files were used to generate the tables and figures presented in the main manuscript. [file 9933219.f1.docx]

This document contains a portion of the analyses performed using SPSS. The analyses have been presented in tabular form in the manuscript. As the original output is not suitable for submission in the system, it has been removed from the system upon your request.

| **Case Processing Summary** | | | | | | | |
| --- | --- | --- | --- | --- | --- | --- | --- |
|  | BMIGROUP | Cases | | | | | |
|  |  | Valid | | Missing | | Total | |
|  |  | N | Percent | N | Percent | N | Percent |
| YAŞ | NON-OBESE | 77 | 96,3% | 3 | 3,8% | 80 | 100,0% |
|  | OBESE | 572 | 98,3% | 10 | 1,7% | 582 | 100,0% |
|  | M-OBESE | 362 | 98,1% | 7 | 1,9% | 369 | 100,0% |
| OGTT0 | NON-OBESE | 77 | 96,3% | 3 | 3,8% | 80 | 100,0% |
|  | OBESE | 572 | 98,3% | 10 | 1,7% | 582 | 100,0% |
|  | M-OBESE | 362 | 98,1% | 7 | 1,9% | 369 | 100,0% |
| OGTT120 | NON-OBESE | 77 | 96,3% | 3 | 3,8% | 80 | 100,0% |
|  | OBESE | 572 | 98,3% | 10 | 1,7% | 582 | 100,0% |
|  | M-OBESE | 362 | 98,1% | 7 | 1,9% | 369 | 100,0% |
| A1C | NON-OBESE | 77 | 96,3% | 3 | 3,8% | 80 | 100,0% |
|  | OBESE | 572 | 98,3% | 10 | 1,7% | 582 | 100,0% |
|  | M-OBESE | 362 | 98,1% | 7 | 1,9% | 369 | 100,0% |
| GFR | NON-OBESE | 77 | 96,3% | 3 | 3,8% | 80 | 100,0% |
|  | OBESE | 572 | 98,3% | 10 | 1,7% | 582 | 100,0% |
|  | M-OBESE | 362 | 98,1% | 7 | 1,9% | 369 | 100,0% |
| AST | NON-OBESE | 77 | 96,3% | 3 | 3,8% | 80 | 100,0% |
|  | OBESE | 572 | 98,3% | 10 | 1,7% | 582 | 100,0% |
|  | M-OBESE | 362 | 98,1% | 7 | 1,9% | 369 | 100,0% |
| ALT | NON-OBESE | 77 | 96,3% | 3 | 3,8% | 80 | 100,0% |
|  | OBESE | 572 | 98,3% | 10 | 1,7% | 582 | 100,0% |
|  | M-OBESE | 362 | 98,1% | 7 | 1,9% | 369 | 100,0% |
| CRP | NON-OBESE | 77 | 96,3% | 3 | 3,8% | 80 | 100,0% |
|  | OBESE | 572 | 98,3% | 10 | 1,7% | 582 | 100,0% |
|  | M-OBESE | 362 | 98,1% | 7 | 1,9% | 369 | 100,0% |
| INS | NON-OBESE | 77 | 96,3% | 3 | 3,8% | 80 | 100,0% |
|  | OBESE | 572 | 98,3% | 10 | 1,7% | 582 | 100,0% |
|  | M-OBESE | 362 | 98,1% | 7 | 1,9% | 369 | 100,0% |
| HOMA | NON-OBESE | 77 | 96,3% | 3 | 3,8% | 80 | 100,0% |
|  | OBESE | 572 | 98,3% | 10 | 1,7% | 582 | 100,0% |
|  | M-OBESE | 362 | 98,1% | 7 | 1,9% | 369 | 100,0% |
| NONHDL | NON-OBESE | 77 | 96,3% | 3 | 3,8% | 80 | 100,0% |
|  | OBESE | 572 | 98,3% | 10 | 1,7% | 582 | 100,0% |
|  | M-OBESE | 362 | 98,1% | 7 | 1,9% | 369 | 100,0% |

| **Descriptives** | | | | | |
| --- | --- | --- | --- | --- | --- |
|  | BMIGROUP | | | Statistic | Std. Error |
| YAŞ | NON-OBESE | Mean | | 33,57 | 1,114 |
|  |  | 95% Confidence Interval for Mean | Lower Bound | 31,35 |  |
|  |  |  | Upper Bound | 35,79 |  |
|  |  | 5% Trimmed Mean | | 33,36 |  |
|  |  | Median | | 34,00 |  |
|  |  | Variance | | 95,564 |  |
|  |  | Std. Deviation | | 9,776 |  |
|  |  | Minimum | | 18 |  |
|  |  | Maximum | | 59 |  |
|  |  | Range | | 41 |  |
|  |  | Interquartile Range | | 17 |  |
|  |  | Skewness | | ,087 | ,274 |
|  |  | Kurtosis | | -,726 | ,541 |
|  | OBESE | Mean | | 36,63 | ,466 |
|  |  | 95% Confidence Interval for Mean | Lower Bound | 35,71 |  |
|  |  |  | Upper Bound | 37,54 |  |
|  |  | 5% Trimmed Mean | | 36,21 |  |
|  |  | Median | | 36,00 |  |
|  |  | Variance | | 124,235 |  |
|  |  | Std. Deviation | | 11,146 |  |
|  |  | Minimum | | 18 |  |
|  |  | Maximum | | 69 |  |
|  |  | Range | | 51 |  |
|  |  | Interquartile Range | | 17 |  |
|  |  | Skewness | | ,437 | ,102 |
|  |  | Kurtosis | | -,371 | ,204 |
|  | M-OBESE | Mean | | 39,60 | ,657 |
|  |  | 95% Confidence Interval for Mean | Lower Bound | 38,31 |  |
|  |  |  | Upper Bound | 40,89 |  |
|  |  | 5% Trimmed Mean | | 39,35 |  |
|  |  | Median | | 39,00 |  |
|  |  | Variance | | 156,057 |  |
|  |  | Std. Deviation | | 12,492 |  |
|  |  | Minimum | | 18 |  |
|  |  | Maximum | | 68 |  |
|  |  | Range | | 50 |  |
|  |  | Interquartile Range | | 20 |  |
|  |  | Skewness | | ,193 | ,128 |
|  |  | Kurtosis | | -,751 | ,256 |
| OGTT0 | NON-OBESE | Mean | | 92,03 | 1,138 |
|  |  | 95% Confidence Interval for Mean | Lower Bound | 89,76 |  |
|  |  |  | Upper Bound | 94,29 |  |
|  |  | 5% Trimmed Mean | | 91,56 |  |
|  |  | Median | | 92,00 |  |
|  |  | Variance | | 99,710 |  |
|  |  | Std. Deviation | | 9,985 |  |
|  |  | Minimum | | 71 |  |
|  |  | Maximum | | 125 |  |
|  |  | Range | | 54 |  |
|  |  | Interquartile Range | | 11 |  |
|  |  | Skewness | | ,774 | ,274 |
|  |  | Kurtosis | | 1,387 | ,541 |
|  | OBESE | Mean | | 91,59 | ,465 |
|  |  | 95% Confidence Interval for Mean | Lower Bound | 90,68 |  |
|  |  |  | Upper Bound | 92,51 |  |
|  |  | 5% Trimmed Mean | | 90,92 |  |
|  |  | Median | | 90,00 |  |
|  |  | Variance | | 123,618 |  |
|  |  | Std. Deviation | | 11,118 |  |
|  |  | Minimum | | 60 |  |
|  |  | Maximum | | 167 |  |
|  |  | Range | | 107 |  |
|  |  | Interquartile Range | | 13 |  |
|  |  | Skewness | | 1,538 | ,102 |
|  |  | Kurtosis | | 6,179 | ,204 |
|  | M-OBESE | Mean | | 94,35 | ,871 |
|  |  | 95% Confidence Interval for Mean | Lower Bound | 92,64 |  |
|  |  |  | Upper Bound | 96,06 |  |
|  |  | 5% Trimmed Mean | | 92,82 |  |
|  |  | Median | | 92,00 |  |
|  |  | Variance | | 274,400 |  |
|  |  | Std. Deviation | | 16,565 |  |
|  |  | Minimum | | 61 |  |
|  |  | Maximum | | 248 |  |
|  |  | Range | | 187 |  |
|  |  | Interquartile Range | | 12 |  |
|  |  | Skewness | | 3,800 | ,128 |
|  |  | Kurtosis | | 27,100 | ,256 |
| OGTT120 | NON-OBESE | Mean | | 105,97 | 3,693 |
|  |  | 95% Confidence Interval for Mean | Lower Bound | 98,62 |  |
|  |  |  | Upper Bound | 113,33 |  |
|  |  | 5% Trimmed Mean | | 103,66 |  |
|  |  | Median | | 103,00 |  |
|  |  | Variance | | 1050,341 |  |
|  |  | Std. Deviation | | 32,409 |  |
|  |  | Minimum | | 50 |  |
|  |  | Maximum | | 207 |  |
|  |  | Range | | 157 |  |
|  |  | Interquartile Range | | 34 |  |
|  |  | Skewness | | 1,115 | ,274 |
|  |  | Kurtosis | | 1,483 | ,541 |
|  | OBESE | Mean | | 111,02 | 1,528 |
|  |  | 95% Confidence Interval for Mean | Lower Bound | 108,02 |  |
|  |  |  | Upper Bound | 114,02 |  |
|  |  | 5% Trimmed Mean | | 108,39 |  |
|  |  | Median | | 105,00 |  |
|  |  | Variance | | 1335,625 |  |
|  |  | Std. Deviation | | 36,546 |  |
|  |  | Minimum | | 32 |  |
|  |  | Maximum | | 324 |  |
|  |  | Range | | 292 |  |
|  |  | Interquartile Range | | 40 |  |
|  |  | Skewness | | 1,417 | ,102 |
|  |  | Kurtosis | | 3,934 | ,204 |
|  | M-OBESE | Mean | | 116,07 | 2,204 |
|  |  | 95% Confidence Interval for Mean | Lower Bound | 111,74 |  |
|  |  |  | Upper Bound | 120,40 |  |
|  |  | 5% Trimmed Mean | | 113,02 |  |
|  |  | Median | | 108,00 |  |
|  |  | Variance | | 1758,053 |  |
|  |  | Std. Deviation | | 41,929 |  |
|  |  | Minimum | | 22 |  |
|  |  | Maximum | | 360 |  |
|  |  | Range | | 338 |  |
|  |  | Interquartile Range | | 45 |  |
|  |  | Skewness | | 1,560 | ,128 |
|  |  | Kurtosis | | 4,879 | ,256 |
| A1C | NON-OBESE | Mean | | 5,436 | ,0384 |
|  |  | 95% Confidence Interval for Mean | Lower Bound | 5,360 |  |
|  |  |  | Upper Bound | 5,513 |  |
|  |  | 5% Trimmed Mean | | 5,436 |  |
|  |  | Median | | 5,500 |  |
|  |  | Variance | | ,113 |  |
|  |  | Std. Deviation | | ,3367 |  |
|  |  | Minimum | | 4,6 |  |
|  |  | Maximum | | 6,7 |  |
|  |  | Range | | 2,1 |  |
|  |  | Interquartile Range | | ,4 |  |
|  |  | Skewness | | ,281 | ,274 |
|  |  | Kurtosis | | 1,736 | ,541 |
|  | OBESE | Mean | | 5,544 | ,0157 |
|  |  | 95% Confidence Interval for Mean | Lower Bound | 5,513 |  |
|  |  |  | Upper Bound | 5,574 |  |
|  |  | 5% Trimmed Mean | | 5,534 |  |
|  |  | Median | | 5,500 |  |
|  |  | Variance | | ,142 |  |
|  |  | Std. Deviation | | ,3763 |  |
|  |  | Minimum | | 3,8 |  |
|  |  | Maximum | | 7,9 |  |
|  |  | Range | | 4,1 |  |
|  |  | Interquartile Range | | ,5 |  |
|  |  | Skewness | | ,621 | ,102 |
|  |  | Kurtosis | | 4,076 | ,204 |
|  | M-OBESE | Mean | | 5,736 | ,0272 |
|  |  | 95% Confidence Interval for Mean | Lower Bound | 5,683 |  |
|  |  |  | Upper Bound | 5,790 |  |
|  |  | 5% Trimmed Mean | | 5,698 |  |
|  |  | Median | | 5,700 |  |
|  |  | Variance | | ,267 |  |
|  |  | Std. Deviation | | ,5169 |  |
|  |  | Minimum | | 4,8 |  |
|  |  | Maximum | | 9,1 |  |
|  |  | Range | | 4,3 |  |
|  |  | Interquartile Range | | ,6 |  |
|  |  | Skewness | | 2,050 | ,128 |
|  |  | Kurtosis | | 9,349 | ,256 |
| GFR | NON-OBESE | Mean | | 112,29 | 1,510 |
|  |  | 95% Confidence Interval for Mean | Lower Bound | 109,28 |  |
|  |  |  | Upper Bound | 115,29 |  |
|  |  | 5% Trimmed Mean | | 112,44 |  |
|  |  | Median | | 114,00 |  |
|  |  | Variance | | 175,602 |  |
|  |  | Std. Deviation | | 13,251 |  |
|  |  | Minimum | | 84 |  |
|  |  | Maximum | | 140 |  |
|  |  | Range | | 56 |  |
|  |  | Interquartile Range | | 19 |  |
|  |  | Skewness | | -,206 | ,274 |
|  |  | Kurtosis | | -,534 | ,541 |
|  | OBESE | Mean | | 111,13 | ,634 |
|  |  | 95% Confidence Interval for Mean | Lower Bound | 109,88 |  |
|  |  |  | Upper Bound | 112,37 |  |
|  |  | 5% Trimmed Mean | | 111,78 |  |
|  |  | Median | | 112,00 |  |
|  |  | Variance | | 229,637 |  |
|  |  | Std. Deviation | | 15,154 |  |
|  |  | Minimum | | 46 |  |
|  |  | Maximum | | 145 |  |
|  |  | Range | | 99 |  |
|  |  | Interquartile Range | | 20 |  |
|  |  | Skewness | | -,635 | ,102 |
|  |  | Kurtosis | | ,503 | ,204 |
|  | M-OBESE | Mean | | 109,22 | ,887 |
|  |  | 95% Confidence Interval for Mean | Lower Bound | 107,48 |  |
|  |  |  | Upper Bound | 110,97 |  |
|  |  | 5% Trimmed Mean | | 109,75 |  |
|  |  | Median | | 111,00 |  |
|  |  | Variance | | 284,878 |  |
|  |  | Std. Deviation | | 16,878 |  |
|  |  | Minimum | | 31 |  |
|  |  | Maximum | | 172 |  |
|  |  | Range | | 141 |  |
|  |  | Interquartile Range | | 21 |  |
|  |  | Skewness | | -,555 | ,128 |
|  |  | Kurtosis | | 1,466 | ,256 |
| AST | NON-OBESE | Mean | | 19,18 | ,882 |
|  |  | 95% Confidence Interval for Mean | Lower Bound | 17,43 |  |
|  |  |  | Upper Bound | 20,94 |  |
|  |  | 5% Trimmed Mean | | 18,34 |  |
|  |  | Median | | 18,00 |  |
|  |  | Variance | | 59,835 |  |
|  |  | Std. Deviation | | 7,735 |  |
|  |  | Minimum | | 7 |  |
|  |  | Maximum | | 63 |  |
|  |  | Range | | 56 |  |
|  |  | Interquartile Range | | 7 |  |
|  |  | Skewness | | 2,884 | ,274 |
|  |  | Kurtosis | | 13,192 | ,541 |
|  | OBESE | Mean | | 19,31 | ,355 |
|  |  | 95% Confidence Interval for Mean | Lower Bound | 18,61 |  |
|  |  |  | Upper Bound | 20,01 |  |
|  |  | 5% Trimmed Mean | | 18,43 |  |
|  |  | Median | | 18,00 |  |
|  |  | Variance | | 71,951 |  |
|  |  | Std. Deviation | | 8,482 |  |
|  |  | Minimum | | 9 |  |
|  |  | Maximum | | 120 |  |
|  |  | Range | | 111 |  |
|  |  | Interquartile Range | | 7 |  |
|  |  | Skewness | | 5,576 | ,102 |
|  |  | Kurtosis | | 51,312 | ,204 |
|  | M-OBESE | Mean | | 20,59 | ,675 |
|  |  | 95% Confidence Interval for Mean | Lower Bound | 19,27 |  |
|  |  |  | Upper Bound | 21,92 |  |
|  |  | 5% Trimmed Mean | | 19,04 |  |
|  |  | Median | | 18,00 |  |
|  |  | Variance | | 164,768 |  |
|  |  | Std. Deviation | | 12,836 |  |
|  |  | Minimum | | 9 |  |
|  |  | Maximum | | 195 |  |
|  |  | Range | | 186 |  |
|  |  | Interquartile Range | | 7 |  |
|  |  | Skewness | | 8,208 | ,128 |
|  |  | Kurtosis | | 98,375 | ,256 |
| ALT | NON-OBESE | Mean | | 19,07 | 1,278 |
|  |  | 95% Confidence Interval for Mean | Lower Bound | 16,53 |  |
|  |  |  | Upper Bound | 21,62 |  |
|  |  | 5% Trimmed Mean | | 17,94 |  |
|  |  | Median | | 17,00 |  |
|  |  | Variance | | 125,827 |  |
|  |  | Std. Deviation | | 11,217 |  |
|  |  | Minimum | | 6 |  |
|  |  | Maximum | | 64 |  |
|  |  | Range | | 59 |  |
|  |  | Interquartile Range | | 10 |  |
|  |  | Skewness | | 1,853 | ,274 |
|  |  | Kurtosis | | 3,947 | ,541 |
|  | OBESE | Mean | | 22,26 | ,644 |
|  |  | 95% Confidence Interval for Mean | Lower Bound | 21,00 |  |
|  |  |  | Upper Bound | 23,53 |  |
|  |  | 5% Trimmed Mean | | 20,51 |  |
|  |  | Median | | 18,00 |  |
|  |  | Variance | | 237,289 |  |
|  |  | Std. Deviation | | 15,404 |  |
|  |  | Minimum | | 6 |  |
|  |  | Maximum | | 221 |  |
|  |  | Range | | 215 |  |
|  |  | Interquartile Range | | 11 |  |
|  |  | Skewness | | 5,413 | ,102 |
|  |  | Kurtosis | | 53,622 | ,204 |
|  | M-OBESE | Mean | | 24,72 | 1,099 |
|  |  | 95% Confidence Interval for Mean | Lower Bound | 22,56 |  |
|  |  |  | Upper Bound | 26,88 |  |
|  |  | 5% Trimmed Mean | | 21,78 |  |
|  |  | Median | | 20,00 |  |
|  |  | Variance | | 437,487 |  |
|  |  | Std. Deviation | | 20,916 |  |
|  |  | Minimum | | 6 |  |
|  |  | Maximum | | 251 |  |
|  |  | Range | | 245 |  |
|  |  | Interquartile Range | | 12 |  |
|  |  | Skewness | | 5,453 | ,128 |
|  |  | Kurtosis | | 44,805 | ,256 |
| CRP | NON-OBESE | Mean | | 5,15 | 2,110 |
|  |  | 95% Confidence Interval for Mean | Lower Bound | ,95 |  |
|  |  |  | Upper Bound | 9,36 |  |
|  |  | 5% Trimmed Mean | | 2,77 |  |
|  |  | Median | | 1,90 |  |
|  |  | Variance | | 342,920 |  |
|  |  | Std. Deviation | | 18,518 |  |
|  |  | Minimum | | 0 |  |
|  |  | Maximum | | 163 |  |
|  |  | Range | | 163 |  |
|  |  | Interquartile Range | | 3 |  |
|  |  | Skewness | | 8,366 | ,274 |
|  |  | Kurtosis | | 72,052 | ,541 |
|  | OBESE | Mean | | 5,31 | ,250 |
|  |  | 95% Confidence Interval for Mean | Lower Bound | 4,82 |  |
|  |  |  | Upper Bound | 5,80 |  |
|  |  | 5% Trimmed Mean | | 4,51 |  |
|  |  | Median | | 3,70 |  |
|  |  | Variance | | 35,607 |  |
|  |  | Std. Deviation | | 5,967 |  |
|  |  | Minimum | | 0 |  |
|  |  | Maximum | | 65 |  |
|  |  | Range | | 64 |  |
|  |  | Interquartile Range | | 5 |  |
|  |  | Skewness | | 4,092 | ,102 |
|  |  | Kurtosis | | 27,095 | ,204 |
|  | M-OBESE | Mean | | 8,71 | ,386 |
|  |  | 95% Confidence Interval for Mean | Lower Bound | 7,95 |  |
|  |  |  | Upper Bound | 9,47 |  |
|  |  | 5% Trimmed Mean | | 7,94 |  |
|  |  | Median | | 6,40 |  |
|  |  | Variance | | 54,039 |  |
|  |  | Std. Deviation | | 7,351 |  |
|  |  | Minimum | | 0 |  |
|  |  | Maximum | | 54 |  |
|  |  | Range | | 53 |  |
|  |  | Interquartile Range | | 7 |  |
|  |  | Skewness | | 2,016 | ,128 |
|  |  | Kurtosis | | 5,929 | ,256 |
| INS | NON-OBESE | Mean | | 15,06 | 3,486 |
|  |  | 95% Confidence Interval for Mean | Lower Bound | 8,12 |  |
|  |  |  | Upper Bound | 22,00 |  |
|  |  | 5% Trimmed Mean | | 11,22 |  |
|  |  | Median | | 11,00 |  |
|  |  | Variance | | 935,670 |  |
|  |  | Std. Deviation | | 30,589 |  |
|  |  | Minimum | | 3 |  |
|  |  | Maximum | | 274 |  |
|  |  | Range | | 271 |  |
|  |  | Interquartile Range | | 7 |  |
|  |  | Skewness | | 8,199 | ,274 |
|  |  | Kurtosis | | 70,014 | ,541 |
|  | OBESE | Mean | | 16,13 | ,405 |
|  |  | 95% Confidence Interval for Mean | Lower Bound | 15,34 |  |
|  |  |  | Upper Bound | 16,93 |  |
|  |  | 5% Trimmed Mean | | 15,16 |  |
|  |  | Median | | 14,02 |  |
|  |  | Variance | | 93,675 |  |
|  |  | Std. Deviation | | 9,679 |  |
|  |  | Minimum | | 4 |  |
|  |  | Maximum | | 119 |  |
|  |  | Range | | 115 |  |
|  |  | Interquartile Range | | 9 |  |
|  |  | Skewness | | 3,443 | ,102 |
|  |  | Kurtosis | | 24,977 | ,204 |
|  | M-OBESE | Mean | | 20,39 | ,641 |
|  |  | 95% Confidence Interval for Mean | Lower Bound | 19,13 |  |
|  |  |  | Upper Bound | 21,65 |  |
|  |  | 5% Trimmed Mean | | 19,12 |  |
|  |  | Median | | 17,33 |  |
|  |  | Variance | | 148,602 |  |
|  |  | Std. Deviation | | 12,190 |  |
|  |  | Minimum | | 4 |  |
|  |  | Maximum | | 98 |  |
|  |  | Range | | 94 |  |
|  |  | Interquartile Range | | 12 |  |
|  |  | Skewness | | 2,151 | ,128 |
|  |  | Kurtosis | | 7,397 | ,256 |
| HOMA | NON-OBESE | Mean | | 3,599 | ,9345 |
|  |  | 95% Confidence Interval for Mean | Lower Bound | 1,737 |  |
|  |  |  | Upper Bound | 5,460 |  |
|  |  | 5% Trimmed Mean | | 2,531 |  |
|  |  | Median | | 2,390 |  |
|  |  | Variance | | 67,243 |  |
|  |  | Std. Deviation | | 8,2002 |  |
|  |  | Minimum | | ,5 |  |
|  |  | Maximum | | 73,1 |  |
|  |  | Range | | 72,6 |  |
|  |  | Interquartile Range | | 1,6 |  |
|  |  | Skewness | | 8,225 | ,274 |
|  |  | Kurtosis | | 70,253 | ,541 |
|  | OBESE | Mean | | 3,696 | ,1014 |
|  |  | 95% Confidence Interval for Mean | Lower Bound | 3,496 |  |
|  |  |  | Upper Bound | 3,895 |  |
|  |  | 5% Trimmed Mean | | 3,433 |  |
|  |  | Median | | 3,085 |  |
|  |  | Variance | | 5,881 |  |
|  |  | Std. Deviation | | 2,4251 |  |
|  |  | Minimum | | ,8 |  |
|  |  | Maximum | | 29,6 |  |
|  |  | Range | | 28,8 |  |
|  |  | Interquartile Range | | 2,0 |  |
|  |  | Skewness | | 3,625 | ,102 |
|  |  | Kurtosis | | 26,260 | ,204 |
|  | M-OBESE | Mean | | 4,823 | ,1747 |
|  |  | 95% Confidence Interval for Mean | Lower Bound | 4,480 |  |
|  |  |  | Upper Bound | 5,167 |  |
|  |  | 5% Trimmed Mean | | 4,440 |  |
|  |  | Median | | 3,935 |  |
|  |  | Variance | | 11,044 |  |
|  |  | Std. Deviation | | 3,3233 |  |
|  |  | Minimum | | 1,0 |  |
|  |  | Maximum | | 27,7 |  |
|  |  | Range | | 26,7 |  |
|  |  | Interquartile Range | | 3,0 |  |
|  |  | Skewness | | 2,559 | ,128 |
|  |  | Kurtosis | | 10,044 | ,256 |
| NONHDL | NON-OBESE | Mean | | 130,5195 | 3,79213 |
|  |  | 95% Confidence Interval for Mean | Lower Bound | 122,9668 |  |
|  |  |  | Upper Bound | 138,0722 |  |
|  |  | 5% Trimmed Mean | | 128,9156 |  |
|  |  | Median | | 132,0000 |  |
|  |  | Variance | | 1107,279 |  |
|  |  | Std. Deviation | | 33,27581 |  |
|  |  | Minimum | | 74,00 |  |
|  |  | Maximum | | 232,00 |  |
|  |  | Range | | 158,00 |  |
|  |  | Interquartile Range | | 46,50 |  |
|  |  | Skewness | | ,529 | ,274 |
|  |  | Kurtosis | | ,466 | ,541 |
|  | OBESE | Mean | | 138,2937 | 1,44038 |
|  |  | 95% Confidence Interval for Mean | Lower Bound | 135,4646 |  |
|  |  |  | Upper Bound | 141,1228 |  |
|  |  | 5% Trimmed Mean | | 137,3411 |  |
|  |  | Median | | 136,0000 |  |
|  |  | Variance | | 1186,723 |  |
|  |  | Std. Deviation | | 34,44884 |  |
|  |  | Minimum | | 52,00 |  |
|  |  | Maximum | | 251,00 |  |
|  |  | Range | | 199,00 |  |
|  |  | Interquartile Range | | 45,00 |  |
|  |  | Skewness | | ,423 | ,102 |
|  |  | Kurtosis | | ,236 | ,204 |
|  | M-OBESE | Mean | | 139,5746 | 1,79869 |
|  |  | 95% Confidence Interval for Mean | Lower Bound | 136,0374 |  |
|  |  |  | Upper Bound | 143,1118 |  |
|  |  | 5% Trimmed Mean | | 138,6495 |  |
|  |  | Median | | 136,0000 |  |
|  |  | Variance | | 1171,176 |  |
|  |  | Std. Deviation | | 34,22245 |  |
|  |  | Minimum | | 58,00 |  |
|  |  | Maximum | | 248,00 |  |
|  |  | Range | | 190,00 |  |
|  |  | Interquartile Range | | 48,00 |  |
|  |  | Skewness | | ,440 | ,128 |
|  |  | Kurtosis | | -,068 | ,256 |

| **Percentiles** | | | | | | | | | |
| --- | --- | --- | --- | --- | --- | --- | --- | --- | --- |
|  |  | BMIGROUP | Percentiles | | | | | | |
|  |  |  | 5 | 10 | 25 | 50 | 75 | 90 | 95 |
| Weighted Average(Definition 1) | YAŞ | NON-OBESE | 18,00 | 19,00 | 25,00 | 34,00 | 42,00 | 45,20 | 49,20 |
|  |  | OBESE | 21,00 | 22,00 | 27,00 | 36,00 | 44,00 | 51,00 | 57,00 |
|  |  | M-OBESE | 21,00 | 22,00 | 29,00 | 39,00 | 49,00 | 57,00 | 61,00 |
|  | OGTT0 | NON-OBESE | 76,00 | 80,00 | 85,50 | 92,00 | 96,00 | 104,00 | 113,30 |
|  |  | OBESE | 77,65 | 80,00 | 84,00 | 90,00 | 97,00 | 105,00 | 111,35 |
|  |  | M-OBESE | 77,00 | 80,00 | 86,00 | 92,00 | 98,25 | 111,00 | 116,00 |
|  | OGTT120 | NON-OBESE | 63,70 | 67,80 | 84,50 | 103,00 | 118,50 | 149,40 | 180,20 |
|  |  | OBESE | 63,30 | 72,30 | 88,00 | 105,00 | 128,00 | 152,00 | 176,35 |
|  |  | M-OBESE | 64,00 | 73,00 | 90,00 | 108,00 | 135,00 | 164,70 | 192,85 |
|  | A1C | NON-OBESE | 4,890 | 5,000 | 5,200 | 5,500 | 5,600 | 5,800 | 5,900 |
|  |  | OBESE | 5,000 | 5,100 | 5,300 | 5,500 | 5,800 | 6,000 | 6,100 |
|  |  | M-OBESE | 5,000 | 5,200 | 5,400 | 5,700 | 6,000 | 6,300 | 6,585 |
|  | GFR | NON-OBESE | 87,00 | 93,00 | 103,00 | 114,00 | 121,50 | 131,00 | 133,20 |
|  |  | OBESE | 82,65 | 91,00 | 102,00 | 112,00 | 122,00 | 130,00 | 133,00 |
|  |  | M-OBESE | 78,00 | 86,00 | 99,00 | 111,00 | 120,00 | 129,00 | 131,85 |
|  | AST | NON-OBESE | 11,90 | 12,80 | 15,00 | 18,00 | 22,00 | 26,40 | 33,30 |
|  |  | OBESE | 12,00 | 13,00 | 15,00 | 18,00 | 22,00 | 26,00 | 30,35 |
|  |  | M-OBESE | 12,00 | 13,00 | 15,00 | 18,00 | 22,00 | 28,00 | 35,85 |
|  | ALT | NON-OBESE | 6,90 | 9,00 | 12,00 | 17,00 | 22,00 | 36,20 | 42,40 |
|  |  | OBESE | 9,00 | 11,00 | 14,00 | 18,00 | 25,00 | 37,00 | 45,35 |
|  |  | M-OBESE | 10,00 | 11,00 | 15,00 | 20,00 | 27,00 | 40,00 | 55,85 |
|  | CRP | NON-OBESE | ,20 | ,40 | ,90 | 1,90 | 4,00 | 9,06 | 11,14 |
|  |  | OBESE | ,70 | 1,10 | 1,90 | 3,70 | 6,60 | 10,90 | 15,55 |
|  |  | M-OBESE | 1,50 | 2,20 | 3,80 | 6,40 | 11,10 | 19,34 | 24,39 |
|  | INS | NON-OBESE | 4,20 | 5,28 | 7,44 | 11,00 | 13,96 | 21,06 | 26,67 |
|  |  | OBESE | 6,52 | 7,73 | 10,29 | 14,02 | 19,19 | 27,47 | 31,99 |
|  |  | M-OBESE | 7,31 | 9,11 | 12,61 | 17,33 | 24,43 | 33,93 | 46,31 |
|  | HOMA | NON-OBESE | ,906 | 1,114 | 1,675 | 2,390 | 3,240 | 4,496 | 6,585 |
|  |  | OBESE | 1,453 | 1,626 | 2,290 | 3,085 | 4,335 | 6,447 | 7,696 |
|  |  | M-OBESE | 1,603 | 1,946 | 2,815 | 3,935 | 5,775 | 8,905 | 10,750 |
|  | NONHDL | NON-OBESE | 79,9000 | 84,8000 | 103,5000 | 132,0000 | 150,0000 | 166,4000 | 199,3000 |
|  |  | OBESE | 88,0000 | 96,3000 | 114,0000 | 136,0000 | 159,0000 | 184,0000 | 200,0000 |
|  |  | M-OBESE | 90,0000 | 98,0000 | 113,0000 | 136,0000 | 161,0000 | 187,0000 | 200,0000 |
| Tukey's Hinges | YAŞ | NON-OBESE |  |  | 25,00 | 34,00 | 42,00 |  |  |
|  |  | OBESE |  |  | 27,00 | 36,00 | 44,00 |  |  |
|  |  | M-OBESE |  |  | 29,00 | 39,00 | 49,00 |  |  |
|  | OGTT0 | NON-OBESE |  |  | 86,00 | 92,00 | 96,00 |  |  |
|  |  | OBESE |  |  | 84,00 | 90,00 | 97,00 |  |  |
|  |  | M-OBESE |  |  | 86,00 | 92,00 | 98,00 |  |  |
|  | OGTT120 | NON-OBESE |  |  | 86,00 | 103,00 | 118,00 |  |  |
|  |  | OBESE |  |  | 88,00 | 105,00 | 128,00 |  |  |
|  |  | M-OBESE |  |  | 90,00 | 108,00 | 135,00 |  |  |
|  | A1C | NON-OBESE |  |  | 5,200 | 5,500 | 5,600 |  |  |
|  |  | OBESE |  |  | 5,300 | 5,500 | 5,800 |  |  |
|  |  | M-OBESE |  |  | 5,400 | 5,700 | 6,000 |  |  |
|  | GFR | NON-OBESE |  |  | 103,00 | 114,00 | 121,00 |  |  |
|  |  | OBESE |  |  | 102,00 | 112,00 | 122,00 |  |  |
|  |  | M-OBESE |  |  | 99,00 | 111,00 | 120,00 |  |  |
|  | AST | NON-OBESE |  |  | 15,00 | 18,00 | 22,00 |  |  |
|  |  | OBESE |  |  | 15,00 | 18,00 | 22,00 |  |  |
|  |  | M-OBESE |  |  | 15,00 | 18,00 | 22,00 |  |  |
|  | ALT | NON-OBESE |  |  | 12,00 | 17,00 | 22,00 |  |  |
|  |  | OBESE |  |  | 14,00 | 18,00 | 25,00 |  |  |
|  |  | M-OBESE |  |  | 15,00 | 20,00 | 27,00 |  |  |
|  | CRP | NON-OBESE |  |  | ,90 | 1,90 | 3,80 |  |  |
|  |  | OBESE |  |  | 1,90 | 3,70 | 6,60 |  |  |
|  |  | M-OBESE |  |  | 3,80 | 6,40 | 11,10 |  |  |
|  | INS | NON-OBESE |  |  | 7,47 | 11,00 | 13,84 |  |  |
|  |  | OBESE |  |  | 10,30 | 14,02 | 19,18 |  |  |
|  |  | M-OBESE |  |  | 12,63 | 17,33 | 24,32 |  |  |
|  | HOMA | NON-OBESE |  |  | 1,710 | 2,390 | 3,230 |  |  |
|  |  | OBESE |  |  | 2,290 | 3,085 | 4,330 |  |  |
|  |  | M-OBESE |  |  | 2,820 | 3,935 | 5,770 |  |  |
|  | NONHDL | NON-OBESE |  |  | 104,0000 | 132,0000 | 150,0000 |  |  |
|  |  | OBESE |  |  | 114,0000 | 136,0000 | 159,0000 |  |  |
|  |  | M-OBESE |  |  | 113,0000 | 136,0000 | 161,0000 |  |  |

| **Test Statistics^a,b^** | | | | | | | | | | | | |
| --- | --- | --- | --- | --- | --- | --- | --- | --- | --- | --- | --- | --- |
|  | YAŞ | OGTT0 | OGTT120 | A1C | GFR | AST | ALT | CRP | INS | HOMA | VKI | NONHDL |
| Chi-Square | 21,873 | 7,551 | 6,072 | 47,826 | 3,622 | 2,998 | 10,967 | 133,588 | 77,476 | 73,583 | 798,512 | 4,187 |
| df | 2 | 2 | 2 | 2 | 2 | 2 | 2 | 2 | 2 | 2 | 2 | 2 |
| Asymp. Sig. | ,000 | ,023 | ,048 | ,000 | ,163 | ,223 | ,004 | ,000 | ,000 | ,000 | ,000 | ,123 |
| a. Kruskal Wallis Test | | | | | | | | | | | | |
| b. Grouping Variable: BMIGROUP | | | | | | | | | | | | |

| **Test Statistics^a,b^** | | | | | | | | | | | | |
| --- | --- | --- | --- | --- | --- | --- | --- | --- | --- | --- | --- | --- |
|  | YAŞ | OGTT0 | OGTT120 | A1C | GFR | AST | ALT | CRP | INS | HOMA | VKI | NONHDL |
| Chi-Square | 4,478 | ,592 | 1,576 | 5,588 | ,052 | ,029 | 4,912 | 20,409 | 19,829 | 17,817 | 211,853 | 3,542 |
| df | 1 | 1 | 1 | 1 | 1 | 1 | 1 | 1 | 1 | 1 | 1 | 1 |
| Asymp. Sig. | ,034 | ,442 | ,209 | ,018 | ,820 | ,866 | ,027 | ,000 | ,000 | ,000 | ,000 | ,060 |
| a. Kruskal Wallis Test | | | | | | | | | | | | |
| b. Grouping Variable: BMIGROUP | | | | | | | | | | | | |
